# As Might Be Reasonably Expected

**Published:** 1890-07-05

**Authors:** 


### Passing Topics.

"AS MIGHT BE REASONABLY EXPECTED."

Those interested in such matters may have noticed that for some few weeks past the daily acknowledgment of funds received by the able and untiring secretary of the British Home for Incurables—how few of us are permitted so delightful a continuity!—has been followed by the statement that "this institution does not participate in the distribution of the Hospital Sunday and Saturday Funds, as might be reasonably expected."

Some time ago we had occasion to comment upon the attempt to compel the Sunday Fund to admit the Royal Hospital for Incurables to the list of participators, and we hope we shall not be considered wanting in sympathy with the admirable work carried on by the several Homes for Incurables if we venture to re-assert that few more unreasonable demands could be made than that these institutions should share in a fund collected for the hospitals, and that it would be quite as much to the point if, by way of emphasizing their appeals, the hospitals were gravely to inform the charitable world that they do not share in the subscriptions forwarded in aid of the incurables. If they did make a statement of the kind the inquiry would naturally arise—Why should they? And similarly we ask of the authorities of the British Home for Incurables how they justify their repeated assertion? "As might be reasonably expected!" Expected by whom? One would suppose the institution had been taken by surprise and was overwhelmed with disappointment at an unexpected refusal of help it had a right to count upon. Yet it must be perfectly well-known to all concerned that the constitution of the Hospital Sunday Fund effectually prevents its distributing committee from entertaining the application of an institution whose benefits are conferred by votes, even were they minded to do so.

It would seem as though the radical difference between a home and a hospital was obvious and intelligible enough to prevent all danger of confusion, yet we should be sorry to believe that the sense of dissatisfaction is deliberately assumed from motives of high financial policy—in order to arouse for the charity, by a subtle suggestion of injustice suffered, the sympathy which we cannot doubt is well assured for it without adventitious aid of any kind.

Few tasks are more grateful than to bear testimony to the value and efficient management of useful institutions. The rise and progress of the Royal Hospital for Incurables constitute one of the noblest of the Victorian legends of charity, while the recent revival of the British Home reflects the highest credit upon its managers. There are few hospitals which can show a financial position so sound, or progress so marked, and perhaps it might be urged that under the present rules of the Sunday Fund, even if participation were permissible, the sums awarded to institutions so far removed from financial difficulty would represent no substantial portion of their expenditure. Far more reasonable would it be if the excluded and dissatisfied institutions should arrange for the collection of some common fund to be distributed among themselves. But, alas! we may not earn their gratitude for the suggestion, for supposing it put into practice, would not their troubles begin with the first success? Example would prove contagious, and institutions all and sundry would hasten to send in their claims. He would be a wise man who could decide the grounds upon which institutions claiming to be for the benefit of incurables could be accepted or rejected. Such a fund would indicate prey for homes and organizations of endless variety. Institutions for the aged, the inebriate, and the orphan might all urge pleas it would be difficult to resist. Even homes for youths might plead a capacity for perennial rejuvenescence, and carpers and critics in general would not be altogether out of the swim. Still, if the authorities are not indisposed to prove by their own experience how difficult it is to please everybody, they might give the idea their consideration.

Agricola.

